# Implications of fetal premature atrial contractions: systematic review

**DOI:** 10.1002/uog.26017

**Published:** 2022-12-01

**Authors:** B. B. Bet, J. M. De Vries, J. Limpens, M. Van Wely, E. Van Leeuwen, S. A. Clur, E. Pajkrt

**Affiliations:** ^1^ Department of Obstetrics and Gynaecology, Amsterdam UMC location University of Amsterdam Amsterdam The Netherlands; ^2^ Amsterdam Reproduction and Development Amsterdam The Netherlands; ^3^ Medical Library, Amsterdam UMC location University of Amsterdam Amsterdam The Netherlands; ^4^ Centre for Reproductive Medicine, Amsterdam UMC location University of Amsterdam Amsterdam The Netherlands; ^5^ Department of Pediatric Cardiology, Amsterdam UMC location University of Amsterdam Amsterdam The Netherlands

**Keywords:** adverse pregnancy outcome, congenital heart defect, fetal arrhythmia, premature atrial contractions, supraventricular tachyarrhythmia

## Abstract

**Objective:**

Fetal heart‐rate irregularities occur in 1–2% of pregnancies and are usually caused by premature atrial contractions (PAC). Although PAC are considered benign, they may be associated with cardiac defects and tachyarrhythmia. We aimed to determine the incidence of congenital heart defects (CHDs) and complications in fetuses with PAC.

**Methods:**

This was a systematic review and meta‐analysis conducted in accordance with the PRISMA statement for reporting items for systematic reviews and meta‐analyses. MEDLINE and EMBASE were searched from 1990 to June 2021 to identify studies on fetuses with PAC. The primary outcome was CHD; secondary outcomes were complications using the endpoints supraventricular tachyarrhythmia (SVT), cardiac failure and intrauterine fetal demise. Meta‐analysis of proportions was performed, subdivided into high‐risk and low‐risk populations based on reason for referral. Pooled incidences with 95% CIs were calculated.

**Results:**

Of 2443 unique articles identified, 19 cohort studies including 2260 fetuses were included. The pooled incidence of CHD in fetuses with PAC was 2.8% (95% CI, 1.5–4.1%), when 0.6% is the incidence expected in the general population. The pooled incidence of CHD was 7.2% (95% CI, 3.5–10.9%) in the high‐risk population and 0.9% (95% CI, 0.0–2.0%) in the low‐risk population. SVT occurred in 1.4% (95% CI, 0.6–3.4%) of fetuses diagnosed with PAC. Cardiac failure was described in 16 fetuses (1.4% (95% CI, 0.5–3.5%)), of which eight were CHD‐related. Intrauterine fetal demise occurred in four fetuses (0.9% (95% CI, 0.5–1.7%)) and was related to CHD in two cases.

**Conclusions:**

Our findings suggest that the risk of CHD in fetuses with PAC is 4–5 times higher than that in the general population. CHD was present more frequently in the high‐risk population. Consequently, an advanced ultrasound examination to diagnose PAC correctly and exclude CHD is recommended. Complications of PAC are rare but can result in fetal demise, thus weekly fetal heart‐rate monitoring remains advisable to enable early detection of SVT and to prevent cardiac failure. © 2022 The Authors. Ultrasound in Obstetrics & Gynecology published by John Wiley & Sons Ltd on behalf of International Society of Ultrasound in Obstetrics and Gynecology.

## Abstract 2

Linked article: There is a comment on this article by Carvalho. Click here to view the Editorial.

A video abstract of this article is available online here.

This article's abstract has been translated into Spanish and Chinese. Follow the links from the abstract to view the translations.

## RESUMEN

Implicaciones de las contracciones auriculares prematuras del feto: revisión sistemática

**Objetivo** Las irregularidades de la frecuencia cardíaca del feto se producen en el 1‐2% de los embarazos y suelen estar causadas por contracciones auriculares prematuras (CAP). Aunque las CAP se consideran benignas, pueden asociarse a defectos cardíacos y taquiarritmias. El objetivo fue determinar la frecuencia de cardiopatías congénitas (CC) y las complicaciones en fetos con CAP.

**Métodos** Se realizó una revisión sistemática y un metaanálisis de acuerdo con la declaración PRISMA para la presentación de informes sobre revisiones sistemáticas y metaanálisis. Se realizaron búsquedas en MEDLINE y EMBASE desde 1990 hasta junio de 2021 para identificar estudios sobre fetos con CAP. El resultado primario fue la CC y los resultados secundarios fueron las complicaciones mediante taquiarritmia supraventricular (TSV), insuficiencia cardíaca y muerte fetal intrauterina. Se realizó un metaanálisis de proporciones, subdividido en poblaciones de alto y bajo riesgo en función del motivo de remisión a especialista. Se calcularon las incidencias combinadas con un IC del 95%.

**Resultados** De los 2443 artículos únicos identificados, se incluyeron 19 estudios de cohortes que incluían 2260 fetos. La incidencia conjunta de CC en fetos con CAP fue del 2,8% (IC 95%, 1,5–4,1%), respecto al 0,6% de incidencia esperada en la población general. La incidencia conjunta de CC fue del 7,2% (IC 95%: 3,5–10,9%) en la población de alto riesgo y del 0,9% (IC 95%: 0,0–2,0%) en la población de bajo riesgo. La TSV se produjo en el 1,4% (IC 95%, 0,6–3,4%) de los fetos diagnosticados con CAP. Se describieron insuficiencias cardíacas en 16 fetos (1,4% [IC 95%, 0,5–3,5%]), de los cuales ocho estaban relacionados con CC. La muerte fetal intrauterina se produjo en cuatro fetos (0,9% [IC 95%, 0,5–1,7%]) y estuvo relacionada con CC en dos casos.

**Conclusiones** Los hallazgos sugieren que el riesgo de CC en fetos con CAP es entre 4 y 5 veces superior al de la población general. La CC fue más frecuente en la población de alto riesgo. Por consiguiente, se recomienda un examen ecográfico avanzado para diagnosticar correctamente las CAP y excluir las CC. Las complicaciones de las CAP son raras pero pueden dar lugar a la muerte fetal, por lo que sigue siendo aconsejable el seguimiento semanal de la frecuencia cardíaca del feto para permitir la detección precoz de la TSV y prevenir el fallo cardíaco.

## 摘要

胎儿房性早搏的影响:系统回顾

摘要

**目的** 1‐2%的妊娠会发生胎儿心率不齐，通常由房性早搏(PAC)引起。虽然PAC被认为是良性的，但它可能与心脏缺陷和快速心律失常有关。我们旨在确定PAC胎儿的先天性心脏缺陷(CHD)和并发症的发生率。

**方法** 这是一项按照PRISMA系统回顾和荟萃分析报告项目声明进行的系统回顾和荟萃分析。检索了1990年至2021年6月的MEDLINE和EMBASE，以确定关于PAC胎儿的研究。主要结果为CHD；次要结果为使用室上性快速性心律失常(SVT)、心力衰竭和宫内胎儿死亡等终点的并发症。进行了比例荟萃分析，根据转诊的原因细分为高风险和低风险人群。计算了合并发生率，置信区间95%。

**结果** 在确定的2443篇原创论文中，包含19项队列研究，其中包括2260例胎儿。PAC胎儿CHD的合并发生率为2.8%(95%CI，1.5‐4.1%)，而普通人群的预期发生率则为0.6%。高风险人群中CHD的合并发生率为7.2%(95%CI，3.5‐10.9%)，低风险人群为0.9%(95%CI，0.0‐2.0%)。在诊断患有PAC的胎儿中，1.4%(95%CI，0.6‐3.4%)出现SVT。16例胎儿出现心力衰竭(1.4%(95%CI，0.5‐3.5%))，其中8例与CHD有关。胎儿宫内死亡4例(0.9%(95%CI，0.5‐1.7%))，其中2例与CHD有关。

**结论** 我们的研究结果表明，PAC胎儿出现CHD的风险比普通人群高4‐5倍。在高风险人群中，CHD出现的频率更高。因此，建议采用先进的超声检查来正确诊断PAC并排除CHD。PAC的并发症很少，但可导致胎儿死亡，因此，仍然建议每周进行一次胎儿心率监测，以便能够及早发现SVT并预防心力衰竭。

**CONTRIBUTION**

**What are the novel findings of this work?**

This meta‐analysis suggests that the incidence of congenital heart defects (CHDs) is higher in fetuses with premature atrial contractions (PAC) than in those without, but is still low in the low‐risk population. Complications such as supraventricular tachyarrhythmia (SVT), cardiac failure and intrauterine fetal demise are rare and occur in less than 2% of cases of PAC.

**What are the clinical implications of this work?**

This work supports the current guideline to perform advanced ultrasonography in fetuses with PAC in order to exclude an associated CHD, and to monitor heart rate during pregnancy for the early detection of SVT and prevention of cardiac failure and intrauterine fetal demise.

## INTRODUCTION

Irregularity of the fetal heart rate occurs in 1–2% of pregnancies^1^, ^2^, ^3^. Fetal arrhythmias can be divided into bradycardia, tachycardia and an irregular rhythm^2^, ^4^. Tachycardia and bradycardia may be signs of fetal distress, but may also be associated with a cardiac abnormality^4^, ^5^, ^6^. The most common cause of an irregular heart beat, with a generally normal heart frequency, is fetal premature atrial contractions (PAC)^1^, ^2^, ^5^, ^7^, ^8^, ^9^, ^10^.

PAC are usually benign and are thought to be the result of the physiological immaturity of the fetal conduction system^9^. As the majority of cases of PAC resolve during pregnancy or the first year after birth, they are of minor clinical consequence. Despite this, current guidelines still advise closely monitoring affected fetuses and their mothers^5^, ^11^, ^12^. The diagnosis of PAC is based on pregnant women undergoing advanced ultrasonography and weekly follow‐up of the fetal heart rate by their obstetrician thereafter^11^, ^13^. This protocol is based on the small possibility that PAC are associated with an underlying congenital heart defect (CHD), and that complications such as supraventricular tachyarrhythmia (SVT), cardiac failure or intrauterine fetal demise can occur^5^, ^9^, ^13^, ^14^.

In the current era, one may wonder if this monitoring protocol is appropriate. Pregnant women undergo a standard second‐trimester anomaly scan in many countries, and most fetuses have already been screened for CHD by the time the arrhythmia is detected^1^, ^2^, ^5^. Therefore, the added value of an additional ultrasound scan to detect CHD is unclear. Furthermore, the same generic monitoring protocol is applied in every mother and fetus with an irregular heart beat. It is not known whether the risk of complications depends upon the frequency or pattern of the PAC (solitary, bigeminy, trigeminy or blocked).

This systematic review and meta‐analysis addressed the following research questions: What is the incidence of CHD in pregnancies affected by fetal PAC? What is the incidence of complications in these pregnancies, using SVT, cardiac failure and intrauterine fetal demise as endpoints? Finally, is the pattern of PAC related to complications observed?

## METHODS

This systematic review was performed according to the preferred reporting items for systematic reviews and meta‐analyses (PRISMA) statement^15^. The research protocol was prepublished in the PROSPERO international database for systematic reviews (CRD42020187873).

### Search strategy and eligibility criteria

A medical information specialist (J.L.) conducted a search in MEDLINE (Ovid) and EMBASE from 1990 to 24 June 2021 to identify studies involving fetuses with PAC. The search strategy consisted of controlled terms (i.e. MeSH‐terms in MEDLINE) and free‐text terms for: (1) irregular heart rhythm including premature contractions and (2) the fetal period (antenatal/prenatal). To minimize the risk of missing studies, the search did not include pregnancy outcomes or study types. Animal studies, reviews and conference abstracts were excluded. No language restrictions were applied. The retrieved records were imported into EndNote X9.3.3 (Clarivate, Philadelphia, PA, USA) and duplicate records were removed. Cited and citing references of the included studies were screened for additional relevant publications. The complete search strategy is presented in Appendix S1.

We included prospective and retrospective cohort studies reporting on CHD or SVT as a complication of PAC. The focus of the article could vary (i.e. outcome of fetal arrhythmia or detection of CHD) but the outcomes for fetuses with PAC had to be mentioned separately from other described populations. Furthermore, the minimum cohort size was 25 subjects, the diagnosis had to be made with M‐mode or pulsed‐Doppler ultrasound and structural fetal echocardiography had to have been performed. We excluded studies published before 1990, those with an unclear method for diagnosing PAC and those with unclear follow‐up.

### Study selection

All abstracts were reviewed independently by two authors (B.B.B. and J.M.d.V.). The selection process was noted in Rayyan together with the reason for exclusion^16^. Disagreement over selection was resolved by consensus. The full text of potentially relevant articles was subsequently reviewed by the same two authors. Disagreement over inclusion was again resolved by consensus and, if necessary, by consultation with a third reviewer (E.P.). Articles published in a language other than English were translated by native speakers.

The quality of the included studies and the risk of bias were assessed independently by two authors (B.B.B. and J.M.d.V.), using the methodological characteristics of the Newcastle–Ottawa scale (NOS) for cohort studies^17^. Studies were scored on the three domains of NOS: selection, comparability and outcome. Additionally, we assessed eligible articles on the quality of described methods, cohort size and presentation of results.

### Data extraction

After selection of the eligible articles, data extraction was completed by one reviewer (B.B.B.) using a predesigned data extraction form. The extracted study variables were: first author's name, country, year of publication, inclusion method, study period, PAC diagnostic method, cohort size, mean gestational age at diagnosis, PAC pattern, incidence of foramen ovale aneurysm, resolution rate, gestational age at resolution, frequency of CHD, type of CHD, incidence of tachyarrhythmia transformation, incidence of cardiac failure, incidence of intrauterine fetal demise, prenatal cardiac drug treatment and other congenital anomalies.

We included subjects with PAC or extrasystoles not further specified, as the majority of extrasystoles are of supraventricular origin^3^, ^6^, ^14^. The pattern of PAC was also noted as intermittent, bigeminy, trigeminy or blocked.

The reason for referral and the baseline population from which the subjects were selected were evaluated. If a study selected only subjects referred for an irregular heart rate, or when the reason for referral was not mentioned, the population was characterized as low risk. When additional referral reasons were present, such as maternal medical conditions, use of medication or unfavorable obstetric or family history, the population was characterized as high risk.

The primary outcome was the incidence of CHD, defined as an abnormality in the structure of the fetal heart, in fetuses with PAC. An aneurysm of the foramen ovale was not classified as a heart defect and was described separately. Persistent ductus arteriosus and Type II atrial septal defects were not considered as CHDs. The secondary outcome was the incidence of fetal complications, using SVT, cardiac failure and intrauterine fetal demise as endpoints. SVT was defined as a heart rate above 180 bpm^2^. Cardiac failure was defined as the inability of the fetal heart to deliver adequate blood flow to organs with the accompanying sonographic findings of cardiomegaly and cardiac effusion and/or fetal hydrops^18^. Intrauterine fetal demise was defined as demise from 16 weeks' gestation until birth.

If the methods or results remained unclear, we approached the authors by e‐mail and/or discussed the diagnosis or complication with our pediatric cardiologist (S.A.C.).

### Statistical analysis

When at least 10 studies had data on primary or secondary outcomes, we performed a meta‐analysis of proportions, using the meta and metafor packages in R version 3.6.1 (R Foundation for Statistical Computing, Vienna, Austria)^19^, ^20^. For each included study we calculated the incidence of primary and secondary outcomes with the corresponding 95% CIs. An average estimate of the incidence was calculated using a random‐effects model with an exact binomial CI. A continuity correction was applied if a study had either zero or all events^20^. The *I* ^2^ test of heterogeneity was expressed as a percentage ranging from 0% to 100%, with higher values indicating more heterogeneity. Heterogeneity was considered high if *I* ^2^ exceeded 50%.

To put the incidence of CHD into perspective, we compared the pooled incidence in fetuses with PAC to that in the general population, using the large registry database of EUROCAT 1999–2008^21^.

## RESULTS

### Study selection and characteristics

**Figure 1:**
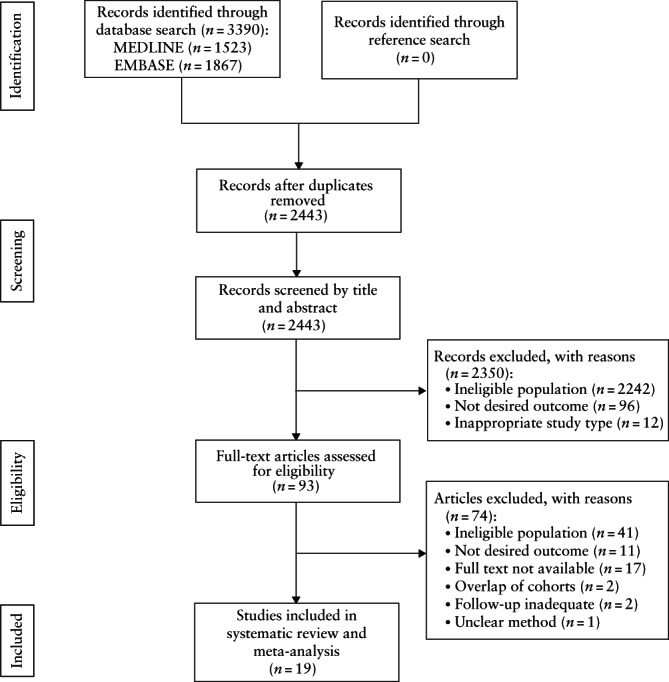
Flowchart summarizing inclusion in systematic review and meta‐analysis of studies evaluating outcome in fetuses with premature atrial contractions.

A total of 2443 articles were identified after duplicates were removed, of which 2350 were excluded based on the title and abstract. Of the remaining 93 articles, 57 were excluded following full‐text assessment because they did not cover the correct population, the method or follow‐up was unclear, the cohort contained fewer than 25 subjects, the cohorts overlapped or the desired outcome was not presented. For a further 17 articles, the full text was not available and the authors could not be reached. Therefore, 19 studies were eventually included in the systematic review and meta‐analysis (Figure 1).

An overview of study characteristics is given in Table 1. Nine studies selected subjects from a high‐risk population^22^, ^23^, ^24^, ^25^, ^26^, ^27^, ^28^, ^29^, ^30^, six selected subjects from a low‐risk population^31^, ^32^, ^33^, ^34^, ^35^, ^36^, and in the remaining four studies the baseline population or reasons for referral were lacking^37^, ^38^, ^39^, ^40^. All studies evaluated cardiac anatomy by fetal echocardiography. Besides M‐mode, Doppler techniques used included color Doppler, continuous‐wave Doppler, pulsed Doppler, Doppler in inferior vena cava, Doppler in vena hepatica and Doppler in pulmonary vein and artery. One study additionally performed fetal magnetocardiography^32^. Four studies, describing a total of 472 fetuses (20.9% of the fetuses included in this review), did not differentiate between atrial and ventricular extrasystoles^26^, ^28^, ^31^, ^32^.

**Table 1:** Characteristics of the 19 included studies evaluating cardiac defects and complications in fetuses with premature atrial contractions (PAC)

Study	Country	Study period	Sample size (*n*)	Population	Diagnostic methods
Boldt (2003)^22^	Finland	1983–2001	200	High risk	Evaluation of cardiac structure; M‐mode, pulsed Doppler, continuous wave Doppler, color Doppler
Calvin (1992)^31^	USA	1987–1990	65	Low risk	Evaluation of cardiac structure; M‐mode, Doppler flow†
Copel (2000)^23^	USA	1985–1995	255	High risk	Evaluation of cardiac structure; M‐mode, color Doppler, pulsed‐wave Doppler in pulmonary artery and vein
Cuneo (2006)^32^	USA	1996–2004	299	Low risk	Evaluation of cardiac structure; M‐mode, pulsed Doppler, fMCG†
Eronen (1997)^24^	Finland	1983–1995	68	High risk	Evaluation of cardiac structure; M‐mode, pulsed Doppler, continuous wave Doppler
Fesslova (2003)^25^	Italy	1984–2002	190	High risk	Evaluation of cardiac structure; M‐mode, color Doppler (since 1986)
Ludwig (2009)^37^	Germany	1993–2005	77	ND	Evaluation of cardiac structure; M‐mode, color Doppler
Maragnes (1991)^38^	Canada	1986–1990	66	ND	Evaluation of cardiac structure; M‐mode, pulsed Doppler
Martin (1990)^26^	USA	1982–1988	58	High risk	Evaluation of cardiac structure; M‐mode, pulsed Doppler†
Oberhänsli (1993)^27^	Switzerland	1986–1991	74	High risk	Evaluation of cardiac structure; M‐mode, pulsed Doppler, color Doppler
Rasiah (2011)^33^	UK	1997–2004	126	Low risk	Evaluation of cardiac structure; M‐mode, pulsed Doppler, color Doppler
Respondek (1997)^28^	Poland	1991–1995	50	High risk	Evaluation of cardiac structure; M‐mode, Doppler techniques not further described†
Saemundsson (2011)^34^	Sweden	8‐year period	228	Low risk	Evaluation of cardiac structure; M‐mode, pulsed Doppler, color Doppler, hepatic venous Doppler
Sivanandam (2011)^35^	USA	2005–2010	67	Low risk	Evaluation of cardiac structure; M‐mode, pulsed Doppler, color Doppler, pulsed‐wave Doppler in pulmonary artery and vein
Trigo (1995)^36^	Portugal	1989–1993	43	Low risk	Evaluation of cardiac structure; M‐mode, pulsed Doppler, color Doppler
Tulzer (1994)^29^	Germany	ND	120	High risk	Evaluation of cardiac structure; M‐mode, simultaneous Doppler, Doppler in IVC
Vergani (2005)^39^	Italy	1985–2002	87	ND	Evaluation of cardiac structure; M‐mode, pulsed Doppler, color Doppler
Wloch (2003)^40^	Poland	1995–2002	122	ND	Evaluation of cardiac structure; M‐mode, Doppler
Zhao (2004)^30^	China	1996–2003	65	High risk*	Evaluation of cardiac structure; M‐mode, pulsed Doppler, color Doppler

Only first author is given for each study.

* Only included PAC with frequency > 1/10 beats.

† Atrial and ventricular extrasystole not differentiated. fMCG, fetal magnetocardiography; IVC, inferior vena cava; ND, not described.

All studies scored four or five stars for risk of bias according to NOS^17^, with two out of four stars for the selection criteria and two or three out of three stars for the outcome criteria (Table S1). The comparability section was not included in our quality assessment since none of the studies used a control group.

The results of the 19 included studies involving 2260 fetuses are presented in Table 2. Sample size ranged from 43 to 299 fetuses. Gestational age at diagnosis was described for fetuses with PAC in six studies, with a mean in the third trimester of between 28 and 33 weeks^22^, ^27^, ^29^, ^36^, ^37^, ^39^. Additionally, six studies described the pattern of PAC^22^, ^27^, ^34^, ^36^, ^38^, ^40^. Of the 733 fetuses in these studies, the pattern was bigeminal or trigeminal in 44 (6.0%). The studies of Maragnes *et al*.^38^ and Wloch *et al*.^40^ accounted for 20 and 15 of these fetuses, respectively. Blocked PAC were noted in 42/733 (5.7%) fetuses, the majority of which were described by Boldt *et al*.^22^ and Oberhänsli *et al*.^27^, with 21 and 15 fetuses, respectively.

**Table 2:** Clinical characteristics and outcomes of 2260 fetuses with premature atrial contractions (PAC)

Study	Subjects	GA at diagnosis (weeks)	Pattern of PAC	CHD	Aneurysmal foramen ovale	Development of SVT	Cardiac failure	Prenatal medication	IUFD	Resolved spontaneously at birth	Neonatal death	Other abnormality
Boldt (2003)^22^	200	32.1 (19–41)	Bigeminy* (7), blocked (21)	18	—	0	3†	7	0	—	7§	Chromosomal anomaly (1)
Calvin (1992)^31^	65	—	—	0	—	0	0	—	—	—	—	ND
Copel (2000)^23^	255	—	—	0	—	0	0	—	0	—	0	ND
Cuneo (2006)^32^	299	—	—	4	—	1	—	—	—	289 (96.7)	—	ND
Eronen (1997)^24^	68	—	—	3	5 (7.4)	—	—	0	0	—	2¶	Trisomy 13 (1)
Fesslova (2003)^25^	190	—	—	14	—	—	5‡	2	0	—	2**	Trisomy 18 (2), trisomy 21 (1), extracardiac anomalies (2)
Ludwig (2009)^37^	77	32.6 ± 4.7	—	15	3 (3.9)	10	—	—	—	53 (68.8)	—	ND
Maragnes (1991)^38^	66	—	Bi/trigeminy (20), blocked (3)	0	—	0	0	—	—	64 (97.0)	—	ND
Martin (1990)^26^	58	—	—	4	—	—	—	—	—	—	—	ND
Oberhänsli (1993)^27^	74	28.5 [17–40]	Blocked (15)	27	—	—	—	—	2	—	5§	Trisomy 18 (2)
Rasiah (2011)^33^	126	—	—	1	—	0	0	0	0	—	0	ND
Respondek (1997)^28^	50	—	—	5	7 (14.0)	4	7	7	0	33 (66.0)	4††	Spina bifida (1)
Saemundsson (2011)^34^	228	—	Bi/trigeminy (2)	2	—	1	—	1	0	225 (98.7)	—	Trisomy 21 (1)
Sivanandam (2011)^35^	67	—	—	0	Not stated (33.3)	0	—	0	—	—	—	ND
Trigo (1995)^36^	43	32 [21–39]	Blocked (1)	0	4 (9.3)	0	0	0	—	—	—	ND
Tulzer (1994)^29^	120	29.9 ± 7.7	—	9	3 (2.5)	—	—	—	2	119 (99.2)	—	Hydrocephalus and omphalocele (1)
Vergani (2005)^39^	87	32 (18.4–41)	—	0	—	2	1	1	0	77 (88.5)	0	ND
Wloch (2003)^40^	122	—	Bi/trigeminy (15), blocked (2)	6	17 (13.9)	4	0	0	—	—	4‡‡	Trisomy 18 (2), plexus choroid cyst (1)
Zhao (2004)^30^	65	—	—	0	—	—	0	0	0	65 (100)	0	ND

Data are given as *n*, median (range), mean ± SD, mean [range] or *n* (%). Only first author is given for each study.

* All received prenatal medication.

† Hydrops developed in combination with congenital heart defect (CHD).

‡ All cases of hydrops/heart failure were associated with CHD, of which two received medication.

§ Not explained further.

¶ One case had trisomy 13 and the other had Ebstein's anomaly.

** One case had trisomy 18 and the other had renal dysplasia.

†† One case was associated with arrhythmia, developed supraventricular tachyarrhythmia (SVT) and hydrops and did not respond to medication.

‡‡ Two cases were associated with CHD; one died of asphyxia and one of neonatal sepsis. GA, gestational age; IUFD, intrauterine fetal demise; ND, none described.

### Fetal outcome

**Figure 2:**
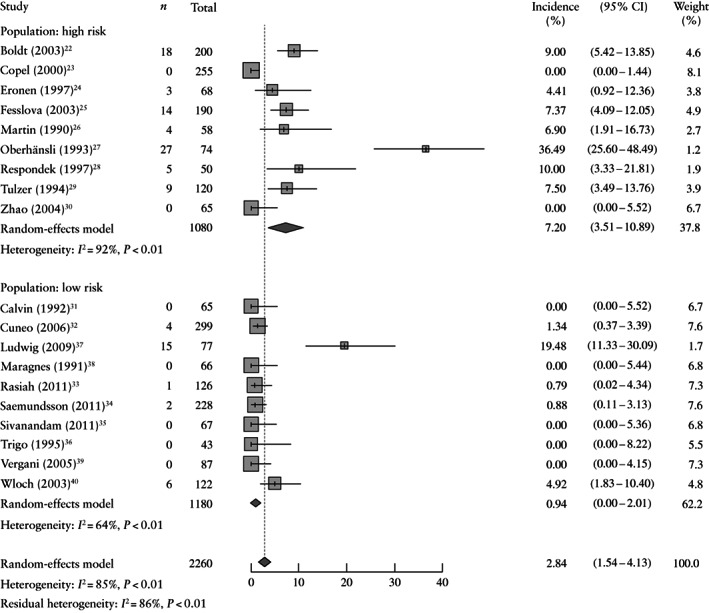
Forest plots showing incidence of congenital heart defect in fetuses with premature atrial contractions, subdivided into high‐risk and low‐risk populations based on reason for referral. Only first author is given for each study.

CHD was found in 108/2260 (4.8%) fetuses with PAC (Table 2). Two studies reported an incidence of CHD higher than 10%^27^, ^37^. Oberhänsli *et al*.^27^ reported the highest incidence of CHD (36.5%). Seven studies found no heart defects in their study population^23^, ^30^, ^31^, ^35^, ^36^, ^38^, ^39^. The meta‐analysis showed a pooled incidence of CHD of 2.8% (95% CI, 1.5–4.1%), with a heterogeneity of 85% (Figure 2). Removing the two outliers^27^, ^37^ decreased the incidence slightly, but it remained in the same range (1.7% (95% CI, 0.8–2.7%)).

Heterogeneity was assessed by dividing the results for studies with high‐risk and low‐risk populations. Nine studies with high‐risk populations showed a pooled incidence of CHD of 7.2% (95% CI, 3.5–10.9%)^22^, ^23^, ^24^, ^25^, ^26^, ^27^, ^28^, ^29^, ^30^ (Figure 2). Ten studies with low‐risk populations (including population not specified) showed a pooled incidence of 0.9% (95% CI, 0.0–2.0%)^31^, ^32^, ^33^, ^34^, ^35^, ^36^, ^37^, ^38^, ^39^, ^40^ (Figure 2). The CIs of the subgroups did not overlap and were significantly different. The heterogeneity was resolved partly by the subgroup analysis, as *I* ^2^ decreased to 64% in the low‐risk population. The impact of the four studies with unclear population was assessed in a sensitivity analysis^37^, ^38^, ^39^, ^40^, which indicated that the results were not affected significantly (pooled incidence, 3.1% (95% CI, 1.5–4.6%)).

The types of CHD detected are shown in Table 3. Among 108 fetuses with PAC and CHD, isolated ventricular septal defect was the most commonly described anomaly (36 cases (33.3%)). One heart defect remained unspecified despite contacting the author^24^.

**Table 3:** Types of congenital heart defect (CHD) reported by 12 studies in which CHD was associated with premature atrial contractions

Study	CHD (*n*)	Type of CHD (*n*)
Boldt (2003)^22^	18	VSD (7), HLHS (2), ToF (2), univentricular heart (2), AVSD (1), cardiac tumor (1), CoA (1), Ebstein's anomaly (1), hypoplasia (1)
Cuneo (2006)^32^	4*	VSD (3), DORV + VSD + pulmonary stenosis (1)
Eronen (1997)^24^	3	VSD (1), Ebstein's anomaly (1), CHD not further described (1)
Fesslova (2003)^25^	14	AVSD (4), ASD (3), pulmonary stenosis (2), dilated right ventricle (1), dysplastic tricuspid valve (non‐Ebstein) (1), myocarditis (1), tricuspid atresia (1), VSD (1)
Ludwig (2009)^37^	15	ASD (5), VSD (4), cardiac tumor (3), tricuspid atresia (1), aortic stenosis (1), pulmonary stenosis + ASD (1)
Martin (1990)^26^	4*	TGA (1), ToF (1), ectopia cordis (1), cardiac tumor (1)
Oberhänsli (1993)^27^	27	VSD (14), AVSD (4), hypertrophic CMP (3), complex heart defect (2), CoA (1), chylothorax (1), tricuspid atresia (1), dilated CMP (1)
Rasiah (2011)^33^	1	VSD (1)
Respondek (1997)^28^	5*	VSD (2), TGA (1), aortic valve stenosis (1), hypertrophic CMP (1)
Saemundsson (2011)^34^	2	VSD (1), AVSD (1)
Tulzer (1994)^29^	9	VSD (2), HLHS (2), aortic stenosis (1), pulmonary atresia (1), TGA + VSD + pulmonary stenosis (1), TGA + aortic atresia (1), tricuspid atresia (1)
Wloch (2003)^40^	6	TGA (1), heterotaxy syndrome (1), AVSD (1), cardiomegaly (2), pericardial effusion (1)

Only first author is given for each study.

* Includes fetuses with atrial or ventricular extrasystole. ASD, atrial septal defect; AVSD, atrioventricular septal defect; CMP, cardiomyopathy; CoA, coarctation of the aorta; DORV, double outlet right ventricle; HLHS, hypoplastic left heart syndrome; TGA, transposition of the great arteries; ToF, tetralogy of Fallot; VSD, ventricular septal defect.

The incidence of CHD in the general population lies between 0.6% and 1.1%^21^, ^41^, ^42^. We used the large cohort of EUROCAT 1999–2008 as a historical benchmark for the incidence of CHD in the general population^21^. Compared with the general population, the incidence of CHD was significantly higher in the whole PAC population (2.8% *vs* 0.6%) and high‐risk population (7.2% *vs* 0.6%), but approximately equal in the low‐risk population (0.9% *vs* 0.6%).

An aneurysm of the foramen ovale was mentioned in seven of the 19 studies^24^, ^28^, ^29^, ^35^, ^36^, ^37^, ^40^ and occurred in 61/547 (11.2% (range, 2.5–33.3%)) fetuses included in these studies.

### Complications

**Figure 3:**
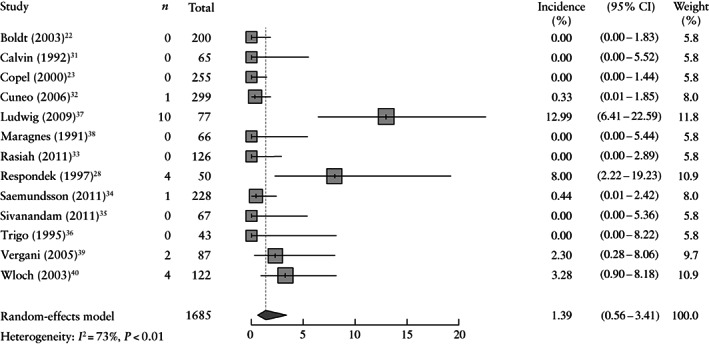
Forest plot showing incidence of supraventricular tachyarrhythmia (SVT) in fetuses with premature atrial contractions. Studies that did not report on SVT were excluded from this meta‐analysis. Only first author is given for each study.

Prenatal SVT was documented as a complication of PAC in 13/19 studies^22^, ^23^, ^28^, ^31^, ^32^, ^33^, ^34^, ^35^, ^36^, ^37^, ^38^, ^39^, ^40^ and occurred in 22/1685 (1.3%) of the included fetuses (Table 2). Two studies reported an incidence of SVT higher than 5%^28^, ^37^, while in seven studies, no fetus developed SVT^22^, ^23^, ^31^, ^33^, ^35^, ^36^, ^38^. The meta‐analysis showed a pooled incidence of 1.4% (95% CI, 0.6–3.4%) for the development of tachyarrhythmia, with a heterogeneity of 73% (Figure 3).

Eleven studies reported cardiac failure or hydrops as an outcome^22^, ^23^, ^25^, ^28^, ^30^, ^31^, ^33^, ^36^, ^38^, ^39^, ^40^. It was observed in 16/1269 (1.3%) fetuses. The study of Respondek *et al*.^28^ reported the highest cardiac failure rate, of 14.0% (7/50 fetuses). In eight out of 16 cases, cardiac failure was due to CHD. The meta‐analysis showed a pooled incidence of 1.4% (95% CI, 0.5–3.5%), with a heterogeneity of 67% (Figure S1). Across 11 studies, 18/1246 (1.4%) fetuses were treated prenatally with cardiac medication, mostly because of SVT, with or without hydrops^22^, ^24^, ^25^, ^28^, ^30^, ^33^, ^34^, ^35^, ^36^, ^39^, ^40^.

Intrauterine fetal demise was reported in four (0.3%) fetuses across 11 studies^22^, ^23^, ^24^, ^25^, ^27^, ^28^, ^29^, ^30^, ^33^, ^34^, ^39^. In the two cases described by Oberhänsli *et al*.^27^, the cause of fetal demise was not noted. In the two cases reported by Tulzer *et al*.^29^, fetal demise was related to hypoplastic left heart syndrome. The meta‐analysis showed a pooled incidence of intrauterine fetal demise of 0.9% (95% CI, 0.5–1.7%) with a heterogeneity of 0% (Figure S2).

### Neonatal outcome

The rate of resolution of PAC before, during or directly after birth was reported in eight studies (Table 2)^28^, ^29^, ^30^, ^32^, ^34^, ^37^, ^38^, ^39^. PAC were reported to have resolved spontaneously in 925/992 (93.2%) fetuses, with resolution rates ranging between 66.0% and 100% across the studies. Neonatal death was described in 24 fetuses^22^, ^23^, ^24^, ^25^, ^27^, ^28^, ^30^, ^33^, ^39^, ^40^, mostly related to cardiac or extracardiac anomalies. Importantly, only one neonate died as a result of tachyarrhythmia and cardiac failure^28^.

## DISCUSSION

This systematic review and meta‐analysis found that the estimated pooled incidence of CHD in pregnancies complicated by fetal PAC was 2.8%, which is 4–5 times higher than the expected incidence in the general population (0.6%)^21^. In the low‐risk subgroup, the incidence of CHD was 0.9% and comparable with that of the general population. Complications of fetal PAC were rare, with an incidence of 1.4% for SVT, 1.4% for cardiac failure and 0.9% for intrauterine fetal demise.

### Strengths and limitations

To the best of our knowledge, this is the first review describing the incidence of CHD in fetuses with PAC. Our intentionally broad search resulted in articles with different focuses but which all included fetuses with PAC as a (sub)group of their study population. The absence of language restriction in the search strategy made it unlikely that important literature was missed. The exclusion of publications before 1990 contributed to greater comparability and reliability of the ultrasound methods used. Additionally, we analyzed complications separately so that we could review specific parts of the current guidelines for the management of fetal PAC.

The study has several limitations which should be acknowledged. First, PAC are an intermittent phenomenon, therefore the true incidence will be higher than the reported incidence, and by inference the true complication rate will also be lower than the reported rate. Second, the study populations differed over time, as CHD screening was initially available only to high‐risk women, so earlier studies included predominantly higher‐risk populations. This selection bias could explain in part the heterogeneity of the results that was demonstrated in the subgroup analysis. In the low‐risk population, the incidence of CHD in fetuses with PAC was found to be comparable with that of the general population. This might suggest that pregnancies that are uneventful except for the occurrence of fetal PAC do not have a higher risk for CHD. As our allocation of fetuses to the low‐risk group when the population type was not defined may have influenced these findings, we performed a sensitivity analysis with the exclusion of those four studies and found that the results were unaffected.

Third, the timing of the diagnosis of CHD is important when interpreting our results. The most interesting cases are those in which CHD was diagnosed after the PAC, as PAC may be the first sign of CHD, thus additional ultrasonography would be indicated. This is particularly relevant for defects observed later in pregnancy (valvular lesions, cardiomyopathies)^5^. Unfortunately, all the studies had a retrospective design and did not report the timing of the CHD diagnosis relative to that of PAC. Consequently, we were unable to assess the value of additional ultrasonography for the detection of CHD in fetuses with PAC. However, in the studies that mentioned the timing of the diagnosis of PAC, it occurred between 28 and 33 weeks' gestation. As first‐ and second‐trimester routine scans are currently offered to pregnant women in many countries, most fetuses would already have been screened for CHD by the time PAC were diagnosed.

Fourth, some cohorts were too small to reliably detect complications of PAC. The incidence of CHD was highest in the small cohort of Oberhänsli *et al*.^27^ (36.5%), and the three largest cohorts reported incidences between 0% and 1.3%^23^, ^32^, ^34^. As no individual study was large enough to reflect a reliable incidence of CHD, we made estimates in our meta‐analysis.

Finally, subtypes of PAC that may be associated with a higher incidence of CHD and SVT (blocked or bigeminal)^14^, ^43^, ^44^, ^45^ were not mentioned in most studies. Reporting bias complicated interpretation of the subtypes risk analysis. In studies with a relatively large number of blocked PAC, the incidence of CHD was also high^22^, ^27^; this requires further investigation. Association of blocked PAC with a higher risk of SVT could not be confirmed owing to the low number of reported cases^43^, ^44^, ^45^. Additionally, PAC were not differentiated from ventricular extrasystoles in all studies, which could have introduced further bias, as ventricular extrasystoles have a higher complication rate than do PAC^2^, ^3^, ^9^.

### Interpretation

In this review, fetal PAC were mostly benign and the majority resolved spontaneously during pregnancy. This is consistent with current fetal medicine guidelines^5^, ^46^. We endorse the recommendation of performing advanced ultrasonography to distinguish PAC correctly from arrhythmias with a higher complication rate and to also (re)assess cardiac anatomy, as we found a higher incidence of CHD among fetuses with PAC. Our findings also support the theory that an aneurysmal foramen ovale may mechanically trigger rhythm disturbances, as it was described in 11.2% of fetuses with PAC^47^, ^48^. Unfortunately, the findings of this review do not help us to determine whether ultrasound assessment is still required in cases in which the first‐/second‐trimester scan was favorable. A specific recommendation for ultrasonography in fetuses with PAC that are frequent, persistent or follow a specific pattern cannot be issued based on our results.

We found the complication rate of fetal PAC to be low. Transformation of PAC to SVT was observed in 1.4% (pooled) of the fetuses in this review, which is comparable with the 0.5–1.0% rate cited in the statement of the American Heart Association^5^, and the advice to perform regular auscultation therefore seems reasonable. The suggestion that certain patterns of PAC increase the risk of SVT and should be monitored more intensively^2^, ^5^ cannot be confirmed or denied by our results, therefore physicians should continue monitoring all fetuses with PAC^3^. Our current protocol to perform weekly monitoring until resolution of PAC is extensive but justified, as it may prevent serious complications^11^. A large prospective cohort study should be conducted in order to determine which PAC patterns warrant close monitoring and to evaluate the additional value of echocardiography after a normal second‐trimester anomaly scan. This would provide the opportunity to truly assess the value of additional echocardiography and to identify risk factors for complications.

### Conclusions

This systematic review supports the current guidelines recommending advanced ultrasonography when fetal PAC are detected. Moreover, follow‐up fetal monitoring should be performed locally for all cases, with clear instructions on when to refer patients back to a fetal medicine unit. However, the low incidence of CHD in a low‐risk population and low complication rate of fetal PAC should be communicated positively to expectant parents and referring physicians.

## Supporting information


**Appendix S1** Complete search strategy

**Table S1** Quality assessment of the included studies using the Newcastle–Ottawa scale for cohort studies

**Figure S1** Forest plot showing incidence of cardiac failure in fetuses with premature atrial contractions. Studies that did not report on cardiac failure were excluded from this meta‐analysis. Only first author is given for each study.

**Figure S2** Forest plot showing incidence of intrauterine fetal demise (IUFD) in fetuses with premature atrial contractions. Studies that did not report on IUFD were excluded from this meta‐analysis. Only first author is given for each study.

## Data Availability

The data supporting these findings of this study are available from the corresponding author upon request.
