# Supplementary material for: Implications of fetal premature atrial contractions: systematic review
Source: Ultrasound Obstet Gynecol. 2022 Dec 1;60(6):721–30. doi: 10.1002/uog.26017 (PMC10107702; doi:10.1002/uog.26017)
Supplement: Supplementary file 1 — Appendix S1 Complete search strategy [file UOG-60-721-s001.docx]

**SUPPLEMENTS**

*Appendix S1 Complete search strategy*

Database(s): **Ovid MEDLINE(R) and Epub Ahead of Print, In-Process, In-Data-Review & Other Non-Indexed Citations and Daily**1946 to June 28, 2021
Search Strategy:**2021-06-29**

| **#** | **Searches** | **Results** |
| --- | --- | --- |
| 1 | arrhythmias, cardiac/ or tachycardia, reciprocating/ or tachycardia, supraventricular/ or tachycardia, ectopic atria/ or tachycardia, ectopic junctional/ or exp cardiac complexes, premature/ | 75696 |
| 2 | (*tachycardia/ or *bradycardia/ or (tachycardia/ or bradycardia/)) and (ultrasonography, prenatal/ or prenatal diagnosis/ or dg.fs. or *fetal diseases/di or ((fetal diseases/ or fetal heart/) and echocardiography/)) | 1360 |
| 3 | (d#srhythm* or arrhythm* or tachy*ythm* or brady*ythm*).tw,kf. | 116740 |
| 4 | (rhythm* adj6 (disorder* or irregular* or abnormal* or anomal* or disturbance* or deviant or (outside adj3 (normal or range)) or chaotic or ectop*)).tw,kf. and (cardiac or heart or ventricul* or atri* or ECG* or echocard* or echo-card*).mp. | 7653 |
| 5 | (((supra-ventric* or supraventr* or ectopic*) adj18 (tachycard* or tachy-card*)) or ((supra-ventric* or supraventr*) adj6 ectop*) or SVT or FSVT).tw,kf. | 10343 |
| 6 | (extrasystol* or extra-systol* or para-systol* or parasystol*).tw,kf. | 4258 |
| 7 | ((prematur* or pre-matur*) adj5 (ventric* or LV or RV or cardiac or atrial) adj2 (complex* or beat* or activit* or contract*)).tw,kf. | 7521 |
| 8 | (((beat* or heartbeat* or atrial or cardiac) adj6 (ectopic or ectopy or ectopies)) not (ectop* adj1 pregnan*)).tw,kf. | 3003 |
| 9 | ((f?etal or f?etus* or prenat* or pre-nat* or antenat* or ante-nat* or in-utero or intrauterin* or intra-uterin*) adj3 (tachycard* or tachy-card* or bradycard* or brady-card*)).ti,kf. | 499 |
| 10 | (atrial adj3 (tachycard* or tachy-card*)).tw,kf. | 5860 |
| 11 | rhythm*.mp. and (tachycard* or tachy-card* or bradycard* or brady-card*).tw,kf. | 9077 |
| **12** | **or/1-11 [arrhythmias]** | **167578** |
| 13 | fetal diseases/ or hydrops fetalis/ | 31363 |
| 14 | heart rate, fetal/ | 5167 |
| 15 | fetal heart/ | 8899 |
| 16 | fetus/ | 79543 |
| 17 | ultrasonography, prenatal/ or prenatal diagnosis/ | 68182 |
| 18 | (f?etus* or f?etal or (hydrops adj3 f?etal*)).tw,kf. | 330996 |
| 19 | (prenat* or pre-nat* or antenat* or ante-nat* or in-utero or intrauterin* or intra-uterin*).tw,kf. | 216972 |
| 20 | (("20*1" or "21*1" or "22*1" or "23*1" or "24*1" or "25*1" or "26*1" or "27*1" or "28*1" or "29*1" or "30*1" or "31*1" or "32*1" or "33*1" or 34*1 or "20th" or "21th" or "22th" or "23th" or "24th" or "25th" or "26th" or "27th" or "28th" or "29th" or "30th" or "31th" or "32th" or "33th" or "34th") adj4 (week* or wk or wks) adj12 gestat*).tw,kf. | 60498 |
| **21** | **or/13-20 [ fetal / prenatal ]** | **543641** |
| **22** | **12 and 21 [ fetal arrhythmia ]** | **3344** |
| 23 | ((exp animals/ or exp animal experimentation/ or models, animal/ or disease models, animal/) not humans/) or (pig or pigs or goat or goats* or sheep or lamb or lambs or ovine or cattle or bovine or cow or cows or horse or horses or mare or calf or calves or dog or dogs or canine or bitch* or cat or cats or feline or rodent* or rabbit* or mice or mouse or murine* or rat or rats or frog or frogs or zebrafish* or fish* or C57BL* or Balb-c or Balbc or wistar or sprague-dawley or dam or dams or pups or pup or ewe or ewes or sow or sows).ti. [EXCLUSION ANIMALS] | 5266743 |
| 24 | editorial/ or "systematic review"/ or cochrane.ti,jw. or (editorial or reply or comment).ti. or ((review/ or (review or overview).ti. or (case or two cases or 2-cases or three cases or 3-cases or four cases or 4-cases).ti. or ((((report* or describ* or (("we" or authors) adj2 present*)) adj4 (case or patient)) or case-presentat* or (case adj2 presented)).tw. and case report*.hw,jw.)) not (cohort studies/ or prospective studies/ or retrospective studies/ or case-control studies/ or "cross-sectional studies"/ or (cohort* or retrospective or prospectiv* or crosssection* or cross-section* or trial or case-serie* or case-control*).ti,ot,kf. or ((chart* or record* or retrospectiv* or cases) adj3 review*).tw,kf.)) | 4592971 |
| **25** | **23 or 24 [exclusion animals/editorials/reviews]** | **9651942** |
| **26** | **22 not 25 [ human fetal arrhythmia - original studies ]** | **2089** |
| **27** | **limit 26 to yr="1990 -Current"** | **1524** |
| **28** | **remove duplicates from 27** | **1523** |

Database(s): **Embase Classic+Embase**1947 to 2021 June 28
Search Strategy: 2021-06-29

| **#** | **Searches** | **Results** |
| --- | --- | --- |
| **1** | **((f?etal or f?etus) adj3 (d#srhythm* or arrhythm* or tachy*ythm* or SVT or brady*ythm* or extrasystol* or extra-systol*)).dq. [ I fetal arrhythmia candidate EMTREE term]** | **73** |
| 2 | heart arrhythmia/ or heart atrium arrhythmia/ or heart supraventricular arrhythmia/ or supraventricular premature beat/ or exp supraventricular tachycardia/ or paroxysmal supraventricular tachycardia/ or extrasystole/ or heart ventricle extrasystole/ or parasystole/ | 179440 |
| 3 | *tachycardia/di or *bradycardia/di or ((*tachycardia/ or *bradycardia/) and (fetus echography/ or fetus electrocardiography/ or *fetus heart/ or *fetus heart rate/ or fetus disease/di or *prenatal diagnosis/)) | 2441 |
| 4 | (d#srhythm* or arrhythm* or tachy*ythm* or brady*ythm*).tw,kw. | 184107 |
| 5 | (rhythm* adj6 (disorder* or irregular* or abnormal* or anomal* or disturbance* or deviant or (outside adj3 (normal or range)) or chaotic or ectop*)).tw,kw. and (cardiac or heart or ventricul* or atri* or ECG* or echocard* or echo-card*).mp. | 13080 |
| 6 | (((supra-ventric* or supraventr* or ectopic*) adj18 (tachycard* or tachy-card*)) or ((supra-ventric* or supraventr*) adj6 ectop*) or SVT or FSVT).tw,kw. | 16649 |
| 7 | (extrasystol* or extra-systol* or para-systol* or parasystol*).tw,kw. | 6985 |
| 8 | ((prematur* or pre-matur*) adj5 (ventric* or LV or RV or cardiac or atrial) adj2 (complex* or beat* or activit* or contract*)).tw,kw. | 11587 |
| 9 | (((beat* or heartbeat* or atrial or cardiac) adj6 (ectopic or ectopy or ectopies)) not (ectop* adj1 pregnan*)).tw,kw. | 4914 |
| 10 | ((f?etal or f?etus* or prenat* or pre-nat* or antenat* or ante-nat* or in-utero or intrauterin* or intra-uterin*) adj3 (tachycard* or tachy-card* or bradycard* or brady-card*)).ti,kw. | 678 |
| 11 | (atrial adj3 (tachycard* or tachy-card*)).tw,kw. | 9975 |
| 12 | rhythm*.mp. and (tachycard* or tachy-card* or bradycard* or brady-card*).tw,kw. | 22003 |
| **13** | **or/2-12 [arrhythmias]** | **291417** |
| 14 | fetus disease/ or prenatal disorder/ | 18311 |
| 15 | fetus hydrops/ | 5580 |
| 16 | fetus heart/ | 7432 |
| 17 | fetus/ | 221851 |
| 18 | fetus electrocardiography/ or fetus heart rate/ or fetus echography/ | 37738 |
| 19 | prenatal diagnosis/ or prenatal screening/ | 67041 |
| 20 | (f?etus* or f?etal or (hydrops adj3 f?etal*)).tw,kw. | 455513 |
| 21 | (prenat* or pre-nat* or antenat* or ante-nat* or in-utero or intrauterin* or intra-uterin*).tw,kw. | 301774 |
| 22 | (("20*1" or "21*1" or "22*1" or "23*1" or "24*1" or "25*1" or "26*1" or "27*1" or "28*1" or "29*1" or "30*1" or "31*1" or "32*1" or "33*1" or 34*1 or "20th" or "21th" or "22th" or "23th" or "24th" or "25th" or "26th" or "27th" or "28th" or "29th" or "30th" or "31th" or "32th" or "33th" or "34th") adj4 (week* or wk or wks) adj12 gestat*).tw,kw. | 88115 |
| **23** | **or/14-22 [ fetal / prenatal ]** | **758181** |
| **24** | **13 and 23 [ II fetal arrhythmia ]** | **5952** |
| **25** | **1 or 24 [ I II fetal arrhythmia ]** | **5962** |
| 26 | ((exp animal/ or animal experiment/ or exp animal model/ or exp experimental animal/ or exp female animal/ or exp veterinary medicine/) not human/) or (pig or pigs or goat or goats* or sheep or lamb or lambs or ovine or cattle or bovine or cow or cows or horse or horses or mare or calf or calves or dog or dogs or canine or bitch* or cat or cats or feline or rodent* or rabbit* or mice or mouse or murine* or rat or rats or frog or frogs or zebrafish* or fish* or C57BL* or Balb-c or Balbc or wistar or sprague-dawley or dam or dams or pups or pup or ewe or ewes or sow or sows).ti. [EXCLUSION ANIMALS] | 6532438 |
| 27 | editorial/ or "systematic review"/ or cochrane.ti,jw. or (editorial or conference abstract or conference review or note).pt. or (editorial or reply or comment).ti. or ((review.pt. or review/ or (review or overview).ti. or (case or two cases or 2-cases or three cases or 3-cases or four cases or 4-cases).ti. or ((((report* or describ* or (("we" or authors) adj2 present*)) adj4 (case or patient)) or case-presentat* or (case adj2 presented)).tw. and case report*.hw,jw.)) not (cohort analysis/ or longitudinal study/ or prospective study/ or retrospective study/ or exp case control study/ or cross-sectional study/ or (cohort* or retrospective or prospectiv* or crosssection* or cross-section* or trial or case-serie* or case-control*).ti,ot,kw. or ((chart* or record* or retrospectiv* or cases) adj3 review*).tw,kw.)) | 10037146 |
| **28** | **26 or 27 [exclusion animals/editorials/reviews]** | **15853139** |
| **29** | **25 not 28 [ human fetal arrhythmia - original studies ]** | **2977** |
| **30** | **limit 29 to yr="1990 -Current"** | **2153** |
| **31** | **remove duplicates from 30 [ human fetal arrhythmia - original studies -deduplicated ]** | **2129** |
| **32** | **31 not medline.cr. [ human fetal arrhythmia - original studies -deduplicated - embase records only ]** | **1867** |
