# Supplementary material for: Implications of fetal premature atrial contractions: systematic review
Source: Ultrasound Obstet Gynecol. 2022 Dec 1;60(6):721–30. doi: 10.1002/uog.26017 (PMC10107702; doi:10.1002/uog.26017)
Supplement: Supplementary file 2 — Table S1 Quality assessment of the included studies using the Newcastle–Ottawa scale for cohort studies [file UOG-60-721-s004.docx]

**Table S1** Quality assessment of the included studies using the Newcastle–Ottawa Scale for cohort studies

|  | **Selection** | | | | **Comparability** | **Outcome** | | | **Stars in total** |
| --- | --- | --- | --- | --- | --- | --- | --- | --- | --- |
|  | Representativeness of exposed cohort | Selection of non-exposed cohort | Ascertainment of exposure | Outcome not present at begin | Comparability | Assessment of the outcome | Length of follow-up | Adequacy of follow-up |  |
| Boldt (2003) | ★ | NA | ★ | NA | NA | ★ | ★ | ★ | 5 |
| Calvin (1992) | ★ | NA | ★ | NA | NA | ★ | ★ | - | 4 |
| Copel (2000) | ★ | NA | ★ | NA | NA | ★ | ★ | - | 4 |
| Cuneo (2006) | ★ | NA | ★ | NA | NA | ★ | ★ | ★ | 5 |
| Eronen (1997) | ★ | NA | ★ | NA | NA | ★ | ★ | ★ | 5 |
| Fesslova (2003) | ★ | NA | ★ | NA | NA | ★ | ★ | ★ | 5 |
| Ludwig (2009) | ★ | NA | ★ | NA | NA | ★ | ★ | ★ | 5 |
| Maragnes (1991) | ★ | NA | ★ | NA | NA | ★ | ★ | - | 4 |
| Martin (1990) | ★ | NA | ★ | NA | NA | ★ | ★ | ★ | 5 |
| Oberhänsli (1993) | ★ | NA | ★ | NA | NA | ★ | ★ | ★ | 5 |
| Rasiah (2011) | ★ | NA | ★ | NA | NA | ★ | ★ | ★ | 5 |
| Respondek (1997) | ★ | NA | ★ | NA | NA | ★ | ★ | ★ | 5 |
| Saemundsson (2011) | ★ | NA | ★ | NA | NA | ★ | ★ | ★ | 5 |
| Sivanandam (2011) | ★ | NA | ★ | NA | NA | ★ | ★ | - | 4 |
| Trigo (1995) | ★ | NA | ★ | NA | NA | ★ | ★ | ★ | 5 |
| Tulzer (1994) | ★ | NA | ★ | NA | NA | ★ | ★ | ★ | 5 |
| Vergani (2005) | ★ | NA | ★ | NA | NA | ★ | ★ | ★ | 5 |
| Wloch (2003) | ★ | NA | ★ | NA | NA | ★ | ★ | - | 4 |
| Zhao (2004) | ★ | NA | ★ | NA | NA | ★ | ★ | ★ | 5 |

NA, not applicable.
