# Supplementary figures and images for: Implications of fetal premature atrial contractions: systematic review
Source: Ultrasound Obstet Gynecol. 2022 Dec 1;60(6):721–30. doi: 10.1002/uog.26017 (PMC10107702; doi:10.1002/uog.26017)

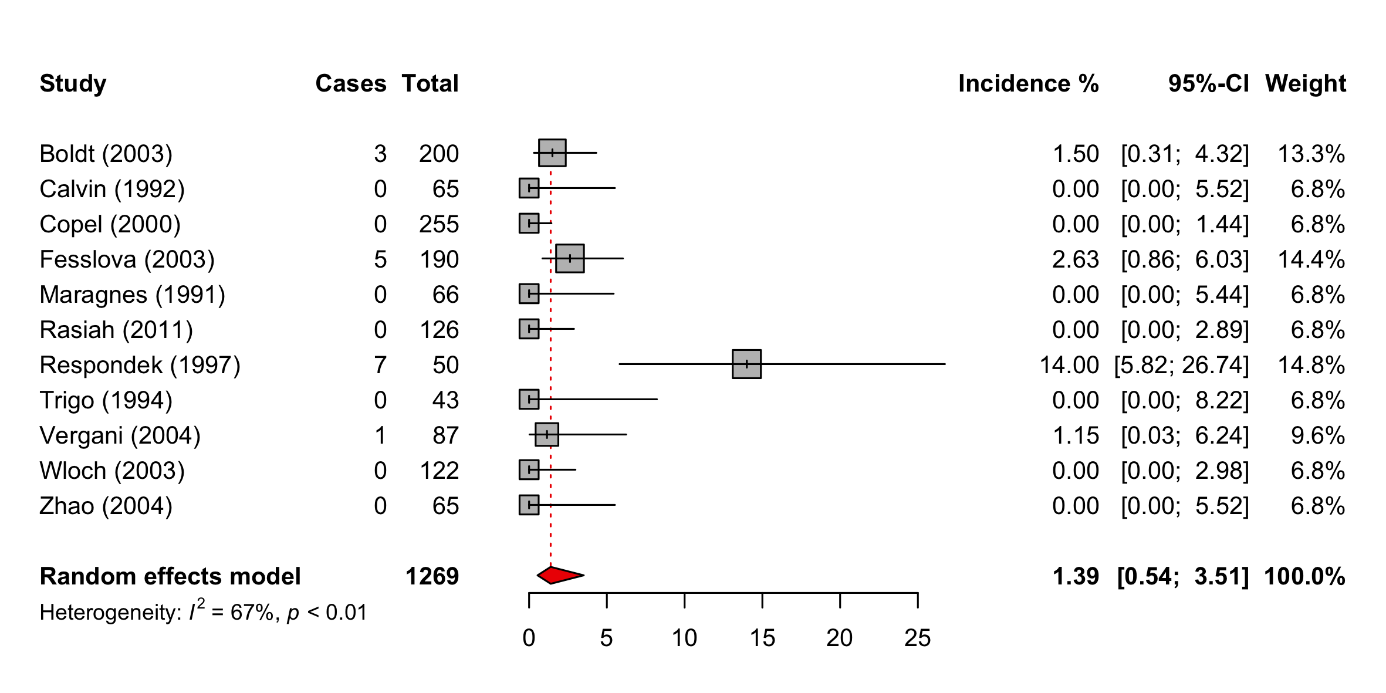

Supplement: Supplementary file 3 — Figure S1 Forest plot showing incidence of cardiac failure in fetuses with premature atrial contractions. Studies that did not report on cardiac failure were excluded from this meta‐analysis. Only first author is given for each study. [file UOG-60-721-s002.tif]

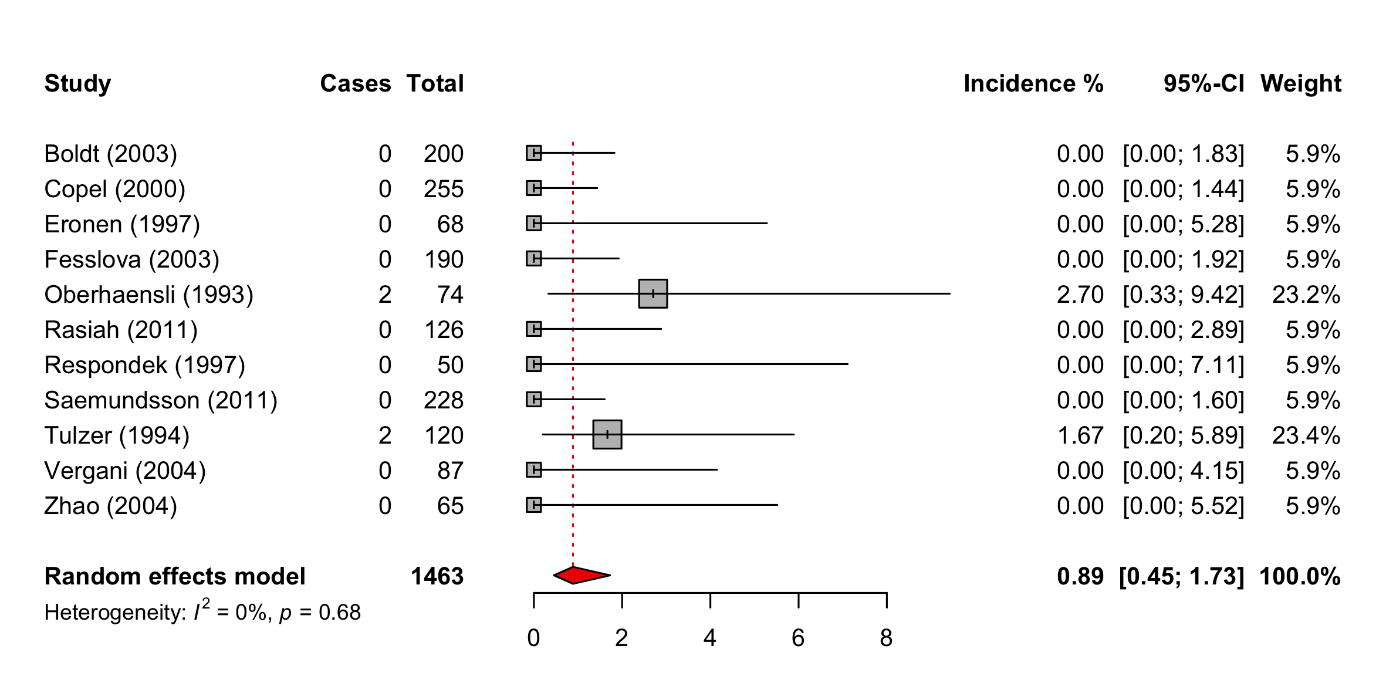

Supplement: Supplementary file 4 — Figure S2 Forest plot showing incidence of intrauterine fetal demise (IUFD) in fetuses with premature atrial contractions. Studies that did not report on IUFD were excluded from this meta‐analysis. Only first author is given for each study. [file UOG-60-721-s003.tif]
